# Supplementary figures and images for: The intergenic small non-coding RNA ittA is required for optimal infectivity and tissue tropism in Borrelia burgdorferi
Source: PLoS Pathog. 2020 May 4;16(5):e1008423. doi: 10.1371/journal.ppat.1008423 (PMC7224557; doi:10.1371/journal.ppat.1008423)

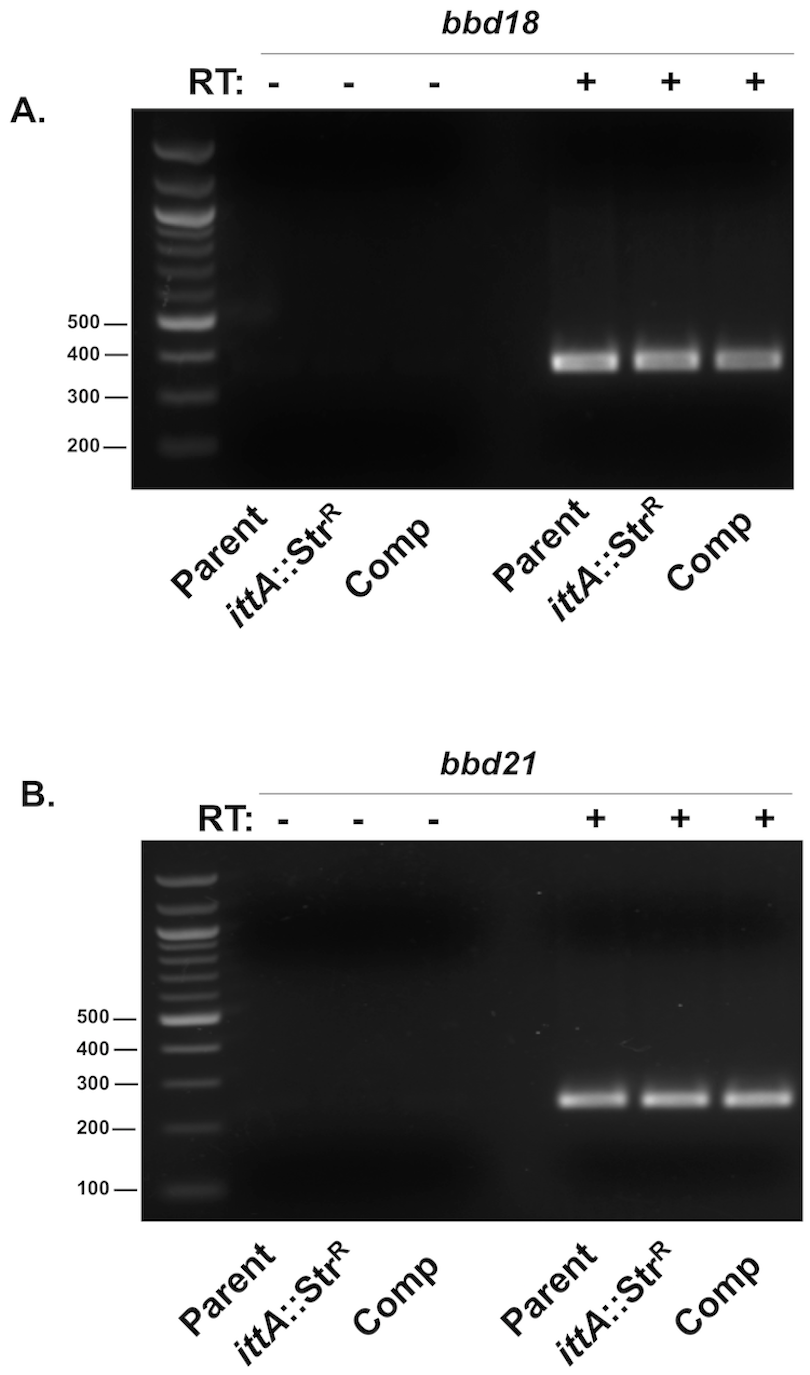

Supplement: S1 Fig — The parent, sRNA mutant (ittA::StrR) and complement (Comp) strains were grown in vitro and total RNA was purified from each. Oligonucleotide primers specific for bbd18 and bbd21 were used without (-) and with (+) added reverse transcriptase (RT). The DNA ladder is shown at the left and the corresponding base pair values are indicated. (TIF) [file ppat.1008423.s010.tif]

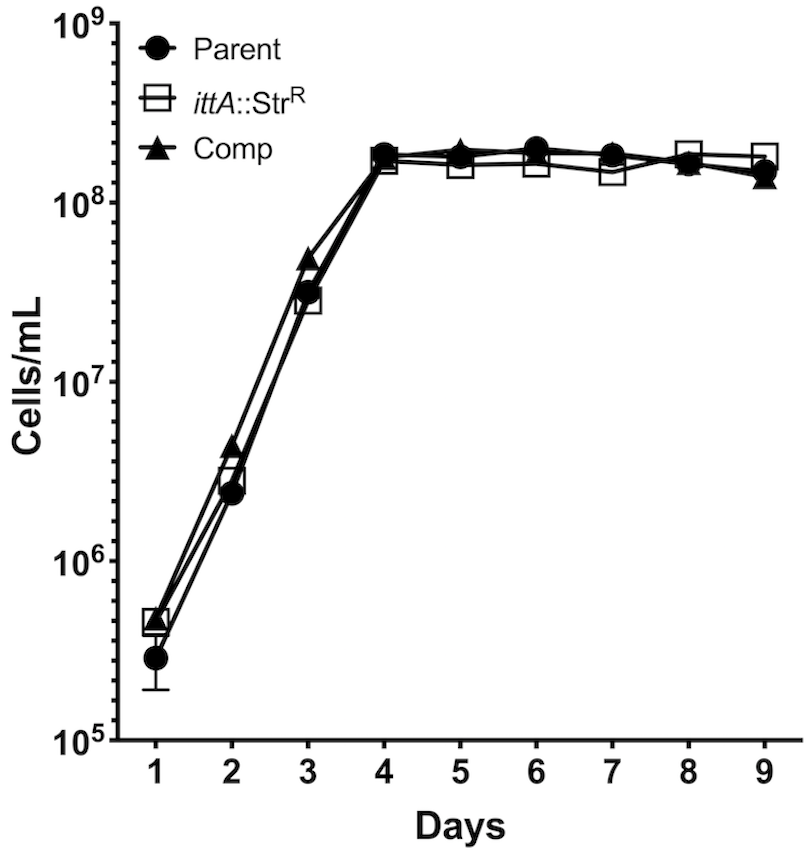

Supplement: S2 Fig — The B. burgdorferi parent strain, the ittA sRNA mutant (ittA::StrR) and the sRNA complement strain (Comp) were grown in conventional microaerophilic conditions of 32°C, 1% CO2, pH 7.6 in triplicate in BSK-II media and enumerated by dark field microscopy daily out to day 9. No significant differences in growth were observed. Similar growth kinetics were observed between these three strains when the cells were grown at conditions of 37°C, 5% CO2 and pH 6.8. Data points shown reflect average value with standard error. (TIF) [file ppat.1008423.s011.tif]

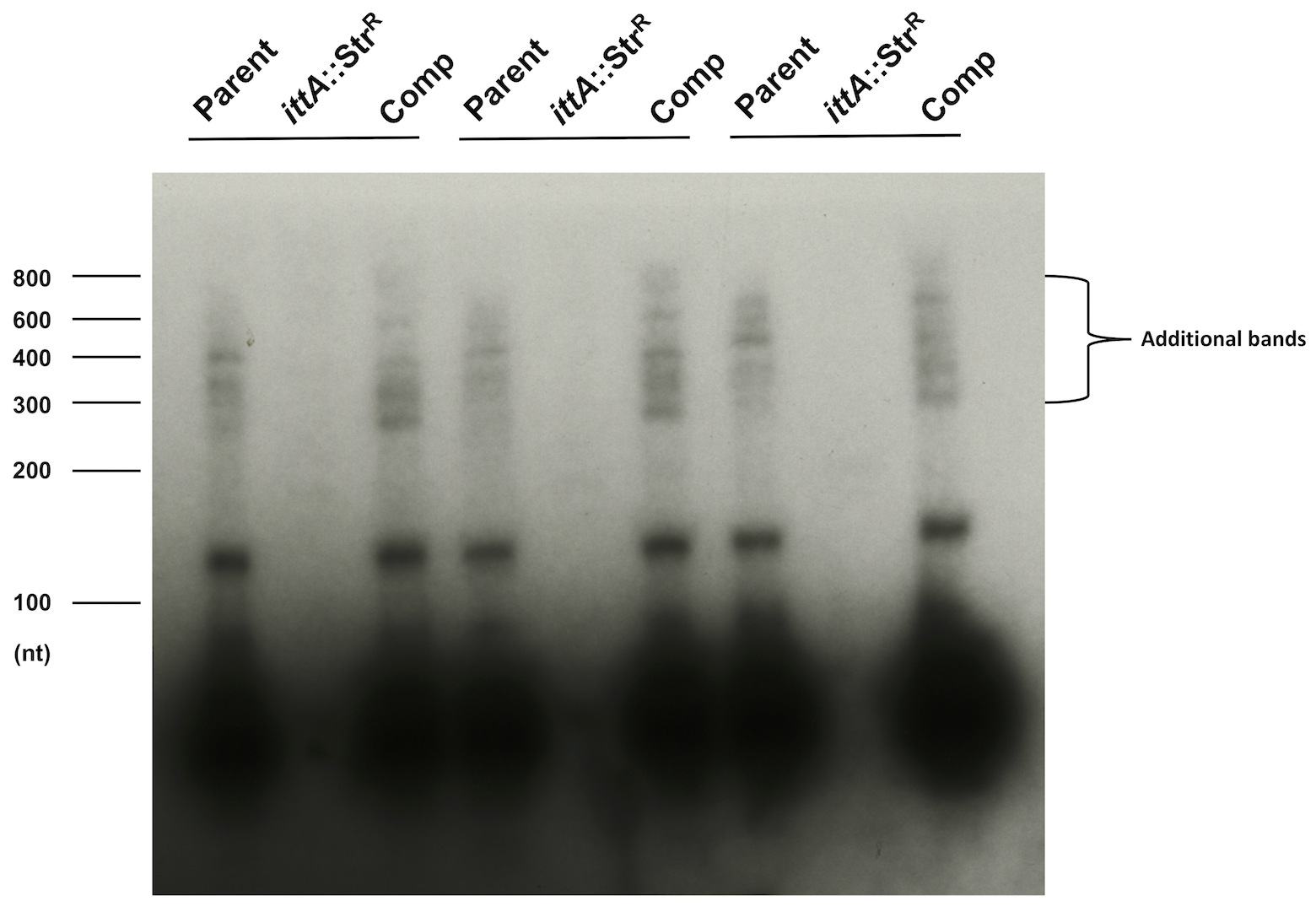

Supplement: S3 Fig — Three biological replicates of the B. burgdorferi parent strain, the sRNA mutant (ittA::StrR) and the sRNA complement strain (Comp), were grown in mammalian-like conditions, RNA was purified and the ittA probe was used for Northern blot analysis at longer exposure. The ittA mutant does not expressed ittA, as expected. The stable processed form of ittA is observed as the dark band underneath the 100 nucleotide marker. The parent and complement strains expression of ittA is comparable, but between 800 and 300 nucleotides, the complement strain exhibits additional bands that are missing in the parent strain and could possibly contribute to the partial complementation of the strain in vitro. The marker is shown in nucleotides at the left of the blot. (TIF) [file ppat.1008423.s012.tif]
